# P49 Evaluation of the national rollout of the training to implement the TARGET stewardship toolkit in primary care in England: a quasi-experimental analysis using hybrid time series modelling

**DOI:** 10.1093/jacamr/dlaf230.056

**Published:** 2025-12-04

**Authors:** Nina Zhu, Ming Xuan Lee, Jade Meadows, Raheelah Ahmad, Donna Lecky

**Affiliations:** Imperial College London, London, UK; UKHSA, UK; UKHSA, UK; City St George's, University of London, London, UK; UKHSA, UK

## Abstract

**Background:**

The UKHSA-led TARGET (Treat Antibiotics Responsibly: Guidance, Education and Tools) antimicrobial stewardship (AMS) toolkit comprises evidence-based resources designed to support primary care clinicians to champion and implement AMS activities; at its core, is the AMS clinical scenario-based training workshop. In October 2022, UKHSA, in collaboration with NHS England (NHSE) initiated the national rollout of the TARGET training programme across NHSE regions through a ‘cascade’ model: from NHSE regional leads to Integrated Care Boards (ICBs), to Primary Care Networks (PCNs) and finally to individual General Practices (GPs).

**Objectives:**

To assess the impact of the national rollout of the TARGET training programme on antibiotic use.

**Methods:**

We extracted the number of antibiotic items dispensed per month (January 2016 to December 2024) from the NHS Business Service Authority (BSA) for each of the 42 ICBs (30 adopters, 12 non-adopters). We calculated the dispensing rate (number of items per 1000 registered patients per month) for England and each ICB and performed linear trend analysis with assessment of autocorrelation. Interrupted time-series analysis (ITSA) using generalized linear models (GLMs) were used to quantify changes in the trend of monthly dispensing rate in the 30 ICBs that adopted the training. Difference-in-differences (DID) analysis with clustered segmented adoption time-series models with fixed-effects, correlated and uncorrelated random-effects estimators, using pooled data from all 42 ICBs were used to estimate the pre-/post-interventional change in monthly dispensing rate, the proportion of broad spectrum antibiotics (cephalosporin, quinolone and co-amoxiclav class) dispensed, the proportion of AWaRe antibiotics dispensed and the trimethoprim to nitrofurantoin dispensing ratio.

**Results:**

Since 2016, the overall background trend of antibiotic use in England was declining at a steady pace, except the upsurge in winter 2022 (due to the national Group A Streptococcal infection outbreak). The ITSA of the 30 adopters showed that most ICBs experienced a reduction in antibiotic dispensing rate after training rollout, but not significant. The DID analysis using fixed-effects estimator suggested that on average, 1.6 less items were dispensed during any month after rollout compared to before (*P*=0.001) amongst the adopters, adjusted for historical trend, seasonality and ICB heterogeneity. We also identified that the duration post-rollout has had a significant, non-linear impact on dispensing rate. It is estimated that there was no change (insignificant increase) in dispensing rate during the first three months immediately after rollout, followed by an accelerating decline (further reduction of 0.02 items per additional month post-rollout, *P*=0.001), indicating a potential delay for the intervention to show effectiveness. The proportion of broad-spectrum antibiotics dispensed was 0.09% less during any month post-rollout (*P*=0.05), further reduced by 0.01% per additional month post-rollout (*P*=0.018). The correlated RE model estimated that across time, the ICBs that adopted training have systematically (insignificantly, *P*=0.773) higher dispensing rate than those without training.

**Conclusions:**

The programme’s timely, budget-conscious delivery using existing infrastructure helped improve antibiotic use, demonstrating its national and international potential for stewardship impact, however regional variation should be acknowledged when implementing similar interventions.

